# Relationship of relative poverty and social relationship on mortality around retirement: a 10-year follow-up of the *Komo-Ise* cohort

**DOI:** 10.1186/s12199-018-0756-6

**Published:** 2018-12-22

**Authors:** Hirokazu Tanaka, Atsushi Miyawaki, Satoshi Toyokawa, Yasuki Kobayashi

**Affiliations:** Department of Public Health, Graduate School of Medicine, The University of Tokyo, 7-3-1 Hongo, Bunkyo-ku, Tokyo, 113-0033 Japan

**Keywords:** Epidemiology, Cohort studies, Socioeconomic factors, Relative poverty, Retirement

## Abstract

**Background:**

As society is aging, retirement takes on increasing importance for individuals in the later life. This study aimed to describe mortality before and after retirement in the Japanese middle-aged/elderly with special attention to socioeconomic position and social relationships.

**Methods:**

We conducted a 10-year follow-up study (the *Komo-Ise* cohort study) and assessed mortality according to socioeconomic positions (relative poverty and occupation) and social relationships (e.g., marital status, living alone, and social support) in workers and the retired. Relative poverty was defined as a household equivalent income of 12,700 US dollars (1.37 million Japanese Yen) or less in 2000. Stratified analyses were conducted according to sex in two groups of employment status: the workers and the retired. Adjusted hazard ratios (HRs) were calculated using the Cox proportional hazard model.

**Results:**

We included 5534 individuals. Of these, 3360 were men (working, 2499; retired, 861) and 2174 were women (working, 1306; retired, 868). We observed 610 deaths (475 in men and 135 in women) during the study period. Relative poverty was a significant risk factor for death (HR 1.52, 95% confidence interval [CI] 1.07–2.14) among retired men but not among working men (HR 1.20, 95% CI 0.79–1.83). Among workers, self-employed men showed a significantly higher hazard of death (HR 1.57, 95% CI 1.09–2.25) than white-collar employees. Retired men who lacked participation in social activities were more likely to die than those who did not (HR 1.44, 95% CI 1.06–1.94). All results, except marital status, indicated non-significant associations in women.

**Conclusions:**

Relative poverty and lack of social engagement may be related to high mortality risk in retired men. Further studies are needed to assess the health status among the middle-aged/elderly population around retirement.

## Introduction

The increasing aging of the population has resulted in a rapid increase in fiscal burden for social security privileges; as such, several governments have responded to this increasing longevity by raising the age of eligibility for senior citizen benefits [1]. Because the age for pension access influences actual retirement age, more workers now need to work beyond the statutory retirement age in economically developed countries, particularly in Japan, which is described as a hyper-aged society. Within this context, a decision of retirement takes on increasing importance for individuals in the later life in modern society.

Previous studies have indicated an association between retirement and mortality and suggested that retirement itself may be related to mortality [2, 3]. Thus, retirement may play an important role as a modifying and/or intermediating factor in the relationship between socioeconomic position/social relationships and mortality. However, prior studies conducted in Japan that investigated the association between relative poverty and relative social deprivation have mainly focused on the retired elderly [4–6]. Saito et al. investigated the impact of social exclusion (including relative poverty) on mortality among Japanese aged 65 years and older (average age was 72.8 years at baseline) [5].

Previous studies have demonstrated an inverse relationship between socioeconomic position and mortality, particularly with income and occupation serving as primary determinants of health [7–9]. Japan faces the concern about inequality in health under a hyper-aged society because the relative poverty rate of older people aged 65 years and over in 2012 was higher than the average in the Organization for Economic Cooperation and Development (OECD) (19.0% vs 12.5%) [1]. The effect of social relationships, such as those between an individual and their spouse or social network, on the risk of mortality is also well established [10, 11]. According to the National Census conducted in 2015, 5,928,000 elderly (65 years and over) were living alone in Japan [12]. Meanwhile, one’s socioeconomic position may change with a major life event such as retirement. Therefore, when considering the transition period between middle and old age, retirement can serve as a major contributor to changes in income and thus, can affect lifestyle, social relationships, physical activity, social contact, and societal roles.

Clearly, income generally decreases, and social engagements change after retirement. Therefore, the lifestyle habits from both before and after retirement should be analyzed separately in the same cohort. In the present study, we aimed to describe mortality among Japanese middle-aged and elderly with a focus on its relationship with socioeconomic position and social relationships stratified by sex and retirement status.

## Methods

### Study participants and design

We used data from the *Komo-Ise* cohort study, which previously revealed that mortality and perceived health were associated with socioeconomic position and degree of social relationships among middle-aged and elderly community dwelling Japanese [13–16]. In addition, the *Komo-Ise* cohort included both middle-aged and elderly individuals who continued to work and who retired across a 7-year period (men and women aged 47–77 years in 2000). Thus, this cohort is suitable for exploring mortality-related risk factors among middle-aged and elderly retired and working men and women.

The *Komo-Ise* cohort consisted of community dwellings men and women residing in Komochi Village and Isesaki City in Gunma Prefecture who were aged 40–69 years in 1993. For the baseline survey, a self-administered questionnaire was administered to 12,630 residents, with a response rate of 91.6% (11,565 residents: 5630 men and 5935 women). The participants who responded to the baseline survey were followed until October 1, 2010. During the follow-up period, follow-up surveys (self-administered questionnaires) were conducted in 2000 and completed by 9533 residents. Of these, participants who had missing information for at least one of the key variables explored here and those who had no history of employment were excluded as the focus of this study was on the effects of retirement.

### Occupations and retirement

**Fig. 1:**
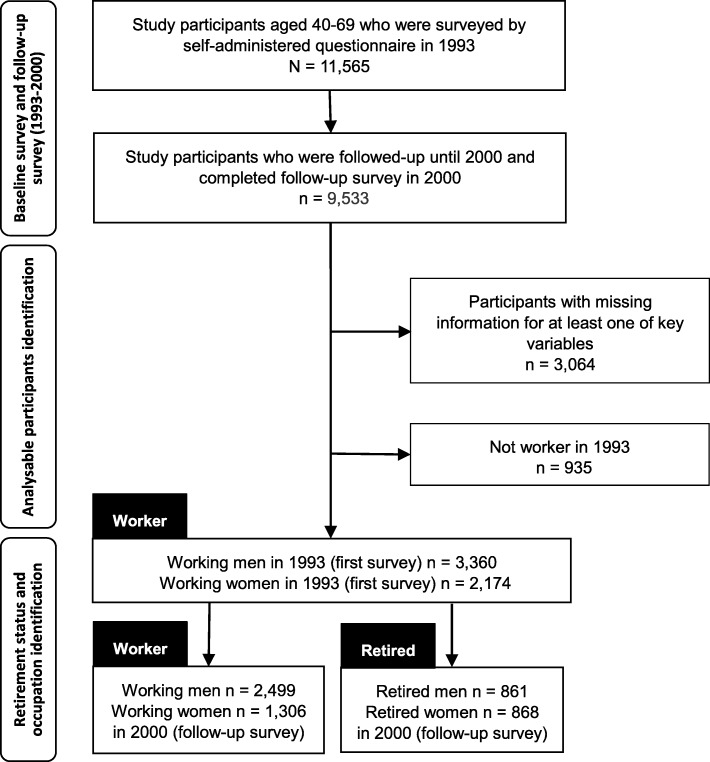
Flow diagram to identify study population: the *Komo-Ise* cohort study

Occupational status was classified into four categories according to responses in the first baseline survey in 1993: white-collar employee, blue-collar employee, self-employed, and agricultural/forestry. In the present study, we defined “retirement” as cessation of work between 1993 and 2000 (e.g., reporting no job in 2000 after having reported a job in 1993) [17]. According to the results of the follow-up survey in 2000 on participant retirement status, we divided men and women into two groups: “working men/women” and “retired men/women.” A flow diagram showing how we identified participants is shown in Fig. 1.

### Ascertaining mortality

Death and migration data in the cohort were identified through resident registration files (*Jumin Kihon Daicho*) from the study region from January 1, 2000 to October 1, 2010 (follow-up period). Participants who had migrated out of the study region were contacted by mail, and non-responders were excluded from the analyses. The average follow-up period was 10.1 years.

### Definition of household equivalent income (relative poverty)

We calculated the household equivalent income using household pre-tax income and family size (number of household members) collected through the survey in 2000. Participants selected their household income from 1 of the 16 categories (one category for every 1 million Japanese yen (JPY) of increase from “0-0.99 million JPY” to “14-14.99 million JPY” and “15 million JPY or over”). Household equivalent income was calculated as follows (in the case of “15 million JPY or over,” 15 million JPY was applied as the income midpoints for that category): household equivalent income = income category midpoint/√ (number of household members). Based on household equivalent income, four income levels were defined to describe income distributions according to the poverty line and the median of household equivalent income among Japanese individuals in 2000, which were derived from the results of the 2000 Comprehensive Survey of Living Conditions [18]. We converted JPY into US dollars (USD) at the rate of 107.8 JPY per USD based on the average exchange rates in 2000 (period average) [19]. The income levels were set and defined as follows: relative poverty: 12,700 USD or less; 12,701–25,400 USD; 25,401–38,100 USD; and more than 38,100 USD. Here, 12,700 USD (1.37 million JPY) was equivalent to the poverty line, and 25,400 USD (2.74 million JPY) was the median of the equivalent household income among Japanese individuals in 2000. This definition of relative poverty was originally from the OECD and was also used in a previous study conducted in Japan [5]. Based on this previous study, we used relative poverty as a socioeconomic position indicator of income level.

### Social relationships

The social relationship score consisted of six items: (1) marital status, (2) living alone, (3) degree of social support, (4) participation in social activity, (5) social isolation, and (6) being bullied. Marital status (1) categories were “married,” “divorced/separated,” or “not married.” Social support (3) was defined according to participant responses to the following question: “Do you have someone to confide in or talk to about yourself or your problems?” Degree of participation in social activities (4) was defined by answers to the following question: “How often do you go out for community or volunteer activities?” Degree of social isolation (5) was defined as having fewer than three close friends or fewer than three close relatives and seeing fewer than three friends or three relatives on a monthly basis. Participants to whom at least two of these conditions applied were classified as being socially isolated. Bullying (6) was defined according to participant response to the following question: “During the past 12 months, were you physically abused by being hit, slapped, pushed, shoved, punched, or threatened with harm by a family member or close friend?” Table 1 shows these questions and the definition of the social relationship score in further detail.

**Table 1:** Items related to social relationships and the classification surveyed in the *Komo-Ise* cohort survey

	Question	Classification (answer)
Marital status	Are you now married, separated, divorced, widowed?	• Married (married)
		• Divorced/separated/widowed (divorced/separated/widowed)
		• Not married (not married)
Living alone	How many people including you live in your households?	• Yes (one) or no
Social support	Someone to confide in or talk to about yourself or your problems	• No (none of the time/a little of the time) or yes
Participation in social activity	How often do you go out for community or volunteer activities?	• No (never) or yes
Social isolation	How many friends or relatives do you feel close to?	• Yes (having fewer than three close friends/having fewer than three close relatives/seeing fewer than three friends or relatives at least once a month)^*^ or no
Bullied	During the past 12 months, were you physically abused by being hit, slapped, pushed, shoved, punched, or threatened with harm by a family member or close friend?	• Yes (yes) or no

*Participants to whom at least two of these applied were classified as being socially isolated

### Statistical analyses

Crude mortality across income levels (relative poverty or not) and occupations was calculated using the person days method. The hazard ratio (HR) for each income level (relative poverty or not) and occupation was estimated using the Cox proportional hazards model. Covariates included age (5-year age categories), region of residence, smoking habits, drinking habits, and perceived health. The HRs of social relationship was also calculated using the Cox proportional hazards model. The occupation was also included as a covariate in the social relationship analyses. These analyses were conducted separately on the two groups (working and retired men/women). All analyses were conducted by sex. We additionally conducted a sensitivity analysis of the income level, using a category boundary of 65 years of age; the multiple-adjusted mortality HRs were estimated among men aged 47–64 and 65–77, respectively. STATA version 15/SE (Stata Corp, College Station, TX, USA) was used for analyses, and a *p* value of < 0.05 was considered significant.

## Results

### Study population

Of the 9533 residents who completed the survey in 2000, we excluded 3064 participants who had missing information for at least one of the key variables explored here and 935 participants who had no history of employment. Finally, 5534 individuals were included. In particular, the cohort comprised 3360 men (working, 2499; retired, 861) and 2174 women (working, 1306; retired, 868). Table 2 shows the distribution of the study population. The average age at the start of the follow-up phase was 58.8 years (standard deviation [SD] 7.5 years) for workers and 67.7 years (SD 6.0 years) for retired men. A total of 37.9% of workers were self-employed, while only 17.8% of the retired men were self-employed. The retired men reported their perceived health status as “fair” or “bad” (54.6%) more frequently than the workers (41.4%) did at baseline survey. From this point forward, we mainly focused our analyses on men only because all results, with the exception of marital status, indicated non-significant associations in women.

**Table 2:** Demographic and socioeconomic characteristics and social relationship of male participants by working status

	Men				Women			
	Workers		Retired		Workers		Retired	
	*n*	%	*n*	%	*n*	%	*n*	%
Total	2499		861		1306		868	
Average age and (SD)	58.8	(7.5)	67.7	(6.0)	57.4	(7.0)	64.6	(6.9)
Age distribution								
47–54 years old	896	35.9	34	4.0	553	42.3	90	10.4
55–59	558	22.3	39	4.5	317	24.3	99	11.4
60–64	398	15.9	156	18.1	206	15.8	214	24.7
65–69	369	14.8	248	28.8	137	10.5	230	26.5
70–74	232	9.3	278	32.3	71	5.4	194	22.4
75–77	46	1.8	106	12.3	22	1.7	41	4.7
Occupation								
White-collar employee	590	23.6	302	35.1	317	24.3	238	27.4
Blue-collar employee	712	28.5	351	40.8	280	21.4	255	29.4
Self-employed	946	37.9	153	17.8	620	47.5	268	30.9
Agricultural/forestry	251	10.0	55	6.4	89	6.8	107	12.3
Region of residence								
Isesaki City (urban)	1423	56.9	580	67.4	856	65.5	599	69.0
Komochi Village (rural)	1076	43.1	281	32.6	450	34.5	269	31.0
Smoking habit								
No	469	18.8	130	15.1	1074	82.2	743	85.6
Current/former	2030	81.2	731	84.9	232	17.8	125	14.4
Drinking habit								
No	1204	48.2	498	57.8	1140	87.3	795	91.6
Yes	1295	51.8	363	42.2	166	12.7	73	8.4
Perceived health status								
Very good	315	12.6	98	11.4	172	13.2	66	7.6
Good	1150	46.0	293	34.0	586	44.9	338	38.9
Fair	978	39.1	405	47.0	527	40.4	423	48.7
Bad	56	2.2	65	7.6	21	1.6	41	4.7
Relative poverty (by household equivalent income)								
Average (SD)	402.2	(214.5)	266.4	(155.0)	398.9	(227.5)	296.7	(192.6)
Yes (≦ 12,700 USD*)	174	7.0	132	15.3	126	9.7	147	16.9
No (12,701–25,400 USD)	655	26.2	428	49.7	331	25.3	347	40.0
No (25,401–38,100 USD)	596	23.9	182	21.1	282	21.6	177	20.4
No (> 38,100 USD)	1074	43.0	119	13.8	567	43.4	197	22.7
Marital status								
Married	2210	88.4	754	87.6	1014	77.6	605	69.7
Divorced/separated	161	6.4	79	9.2	222	17.0	211	24.3
Not married	128	5.1	28	3.3	70	5.4	52	6.0
Living alone								
No	2417	96.7	814	94.5	1221	93.5	757	87.2
Yes	82	3.3	47	5.5	85	6.5	111	12.8
Social support								
Yes	2062	84.3	720	84.9	1223	94.2	803	93.5
No	383	15.7	128	15.1	76	5.9	56	6.5
Participation in social activities								
Yes	882	36.1	325	38.6	398	30.9	311	36.2
No	1559	63.9	517	61.4	891	69.1	548	63.8
Social isolation								
Not isolated	1881	78.2	666	79.6	1049	82.9	710	85.0
Isolated	524	21.8	171	20.4	216	17.1	125	15.0
Bullied								
No	2331	93.8	795	94.9	1136	87.4	773	91.9
Yes	155	6.2	43	5.1	164	12.6	68	8.1

*12,700 US dollars (USD) equaled to 1.37 million Japanese Yen: the poverty line among Japanese in 2000 (Comprehensive Survey of Living Conditions in 2000)

*SD* standard deviation

### Mortality risk across income levels (relative poverty) and occupations

We observed 610 deaths (475 in men and 135 in women) during the study period, whereas 27 persons (17 men and 10 women) were censored due to immigration. Crude mortality, age-adjusted, and multiple-adjusted mortality HRs across income levels (relative poverty or not) and occupations are shown in Table 3. Inverse associations between household equivalent income and crude mortality were observed among workers, although these were non-significant. The multiple-adjusted mortality HR of working men in relative poverty was 1.20 (95% confidence intervals [95% CI] 0.79–1.83) compared to working men whose household equivalent income did not indicate relative poverty. The multiple-adjusted mortality HR of retired men in relative poverty was 1.52 (95% CI 1.07–2.14) compared to retired men whose household equivalent income did not indicate relative poverty. Regarding occupation, the HR of men working in blue-collar positions showed a higher multiple-adjusted hazard risk (1.24, 95% CI 0.82–1.87) than white-collar employees, but the difference was non-significant. The HR of men working in agriculture and forestry displayed a lower multiple-adjusted hazard risk (0.93, 95% CI 0.53–1.62) than that of white-collar employees, although this was also non-significant. Those who were self-employed (workers) had the highest crude mortality and significant multiple-adjusted high HR (1.57, 95% CI 1.09–2.25) compared to white-collar employees. All results indicated non-significant associations in women: relative poverty was not a significant risk factor for death (HR 0.98, 95% CI 0.46–2.06) among working women.

**Table 3:** The mortality across relative poverty and occupation estimated by the Cox proportional hazards model

	Person years	Number of death	Crude mortality^†^	Age adjusted				Multiple-adjusted^⁑^			
				HR	95% CI			HR	95% CI		
Men											
Workers (*n* = 2499)											
Relative poverty (household equivalent income)											
Yes (≦ 12,700 USD*)	1782	24	13.5	1.21	(0.79–1.85)			1.20	(0.79–1.83)		
No (> 12,700 USD *)	23,954	225	9.4	Reference				Reference			
Occupation											
White-collar employee	6093	40	6.6	Reference				Reference			
Blue-collar employee	7431	54	7.3	1.24	(0.82–1.86)			1.24	(0.82–1.87)		
Self-employed	9596	131	13.7	1.60	(1.12–2.29)			1.57	(1.09–2.25)		
Agricultural/forestry	2616	24	9.2	0.76	(0.45–1.27)			0.93	(0.53–1.62)		
Retired (*n* = 861)											
Relative poverty (household equivalent income)											
Yes (≦ 12,700 USD*)	1181	41	34.7	1.47	(1.04–2.08)			1.52	(1.07–2.14)		
No (> 12,700 USD*)	6958	185	26.6	Reference				Reference			
Occupation											
White-collar employee	2875	74	25.7	Reference				Reference			
Blue-collar employee	3384	84	24.8	1.03	(0.76–1.42)			1.00	(0.73–1.37)		
Self-employed	1380	48	34.8	1.46	(1.01–2.10)			1.29	(0.89–1.86)		
Agricultural/forestry	501	20	39.9	1.41	(0.86–2.32)			1.31	(0.75–2.27)		
Women											
Workers (*n* = 1306)											
Relative poverty (household equivalent income)											
Yes (≦ 12,700 USD*)	1320	9	6.8	1.12	(0.53–2.33)			0.98	(0.46–2.06)		
No (> 12,700 USD*)	12,488	44	3.5	Reference				Reference			
Occupation											
White-collar employee	3369	5	1.5	Reference				Reference			
Blue-collar employee	2971	9	3.0	1.85	(0.62–5.52)			1.98	(0.66–5.93)		
Self-employed	6548	33	5.0	1.78	(0.68–4.70)			1.85	(0.69–4.96)		
Agricultural/forestry	921	6	6.5	2.10	(0.62–7.08)			1.84	(0.50–6.78)		
Retired (*n* = 868)											
Relative poverty (household equivalent income)											
Yes (≦ 12,700 USD*)	1543	11	7.1	0.67	(0.36–1.27)			0.56	(0.29–1.07)		
No (> 12,700 USD*)	7451	71	9.5	Reference				Reference			
Occupation											
White-collar employee	2452	21	8.6	Reference				Reference			
Blue-collar employee	2657	23	8.7	0.99	(0.55–1.78)			0.89	(0.49–1.62)		
Self-employed	2761	26	9.4	1.00	(0.56–1.78)			0.91	(0.51–1.63)		
Agricultural/forestry	1123	12	10.7	1.01	(0.50–2.07)			1.09	(0.47–2.53)		

*HR* hazard ratio, *95% CI* 95% confidence intervals

*12,700 US dollars (USD) equaled to 1.37 million Japanese Yen: the poverty line among Japanese in 2000 (Comprehensive Survey of Living Conditions in 2000)

⁑Multiple adjusted models were controlled for age (5-year age categories), area of residence, smoking habit, drinking habit, and perceived health

†Per 1000 person-years

In the sensitivity analysis, the multiple-adjusted mortality HR was 3.00 (95% CI 1.46–6.14) among retired men aged 47–64 years (*n* = 229, average age 59.8 years) and 1.17 (95% CI 0.61–2.24) among working men aged 47–64 years (*n* = 1852, average age 55.2 years). The multiple-adjusted mortality HR was 1.23 (95% CI 0.71–2.16) among working men aged 65–77 (*n* = 647, 69.2 years) and 1.27 (95% CI 0.85–1.93) among retired men aged 65–77 (*n* = 632, 70.5 years). A total of 55.5% retired men aged 47–64 years reported their perceived health status as fair or bad at baseline survey, whereas the percentage was 41.1% among working men aged 47–64 years.

### Mortality risk across social relationships

Table 4 shows the multiple-adjusted mortality HRs between social relationships. Among workers, marital status, and living conditions (living alone) was associated with increased hazard of death. The multiple-adjusted mortality HRs of divorced/separated and unmarried working men were 1.56 (95% CI 1.04–2.33) and 2.02 (95% CI 1.12–3.65), respectively, and were significant when compared to that of married working men. However, that of the divorced/separated men was 0.90, showing a non-significant difference (95% CI 0.57–1.42) from married retired men. Working men who lived alone were more likely to die than those who did not (HR 1.82, 95% CI 1.08–3.08). However, this association was not observed for retired men who lived alone (HR 0.98, 95% CI 0.55–1.76). An increased hazard of death due to lack of participation in social activities was observed among retired men (HR 1.44, 95% CI 1.06–1.94) but not among working men (HR 1.03, 95% CI 0.79–1.35). Being bullied may be associated with increased hazard of death among retired men, although this was non-significant (HR 1.45, 95% CI 0.84–2.50). All results indicated non-significant associations in women, but living alone may be risk factor for death (HR 1.67, 95% CI 0.98–2.86) among retired women.

**Table 4:** The hazard ratios estimated by the Cox proportional hazards model by social relationships*

	Men				Women			
	Workers		Retired		Workers		Retired	
	HR	(95% CI)	HR	(95% CI)	HR	(95% CI)	HR	(95% CI)
Marital status								
Married	Reference		Reference		Reference		Reference	
Divorced/separated	1.56	(1.04–2.33)	0.90	(0.57–1.42)	1.07	(0.54–2.14)	1.25	(0.77–2.04)
Not married	2.02	(1.12–3.65)	2.11	(1.03–4.31)	2.75	(1.09–6.93)	0.73	(0.22–2.39)
Living alone								
No	Reference		Reference		Reference		Reference	
Yes	1.82	(1.08–3.08)	0.98	(0.55–1.76)	1.43	(0.58–3.51)	1.67	(0.98–2.86)
Social support								
Yes	Reference		Reference		Reference		Reference	
No	0.99	(0.69–1.42)	1.18	(0.83–1.69)	1.10	(0.37–3.24)	0.65	(0.26–1.64)
Participation in social activity								
Yes	Reference		Reference		Reference		Reference	
No	1.03	(0.79–1.35)	1.44	(1.06–1.94)	1.04	(0.54–1.97)	1.17	(0.72–1.91)
Social isolation								
Not isolated	Reference		Reference		Reference		Reference	
Isolated	1.03	(0.75–1.40)	0.95	(0.68–1.32)	0.97	(0.44–2.11)	0.73	(0.36–1.46)
Bullied								
No	Reference		Reference		Reference		Reference	
Yes	1.01	(0.59–1.70)	1.45	(0.84–2.50)	1.38	(0.59–3.22)	0.90	(0.35–2.30)

*HR* hazard ratio, *95% CI* 95% confidence intervals

*All estimations were controlled for age (5-year age categories), occupation, area of residence, smoking habit, drinking habit, and perceived health

## Discussion

Relative poverty may be related to an increased risk of mortality in retired men, but not in women. This is a new finding among Japanese populations. A lack in social relationship, such as living alone among workers or lack of participation in social activities, among the retired was related to higher mortality rate. Regarding occupation-related differences, we also found that men working in agriculture and forestry had a lower mortality rate than men working white-collar positions, although the difference was not significant. Self-employed workers had higher mortality rate than white-collar employees. For women, mortality differences by socioeconomic position and social relationships were minimal and/or non-significant regardless of retirement status, except marital status. This finding complements those of previous studies and clarifies the associations between socioeconomic position/social relationships and mortality in both working and the retired people in Japanese community dwellings.

In our analyses, a robust, inverse relationship between income level (in terms of relative poverty) and mortality risk was found only among retired men. However, there are two issues that need to be addressed further. First, Japan is known to have lower health inequality than other developed countries [20]. Our findings suggest that mortality inequality by income levels is limited to working men in Japanese community dwellings. However, mortality risk among retired men with relative poverty was higher than among those who did not experience relative poverty. This finding suggests that relative poverty after retirement should be considered as an important socioeconomic indicator of life expectancy. Second, in our study, because household incomes were defined by an income level classification, relative poverty was not equivalent with individuals’ income. Because retired men themselves do not earn money, their household income is usually derived from their pension and/or the other family members’ earnings. If future pension payments decrease, the disparity in household equivalent income between those who live alone and those who live with their families would likely increase.

Our findings are in contrast to those reported in a previous study that found a non-significant association between mortality and relative poverty among elderly men and women in Japan [5]. A possible explanation for the high mortality risk among retired individuals in relative poverty found in the current study is that the study population was younger (average age in men was 67.7 years at baseline) than that in the previous study [5]. Our additional analysis (sensitivity analysis) showed that relative poverty was significantly associated with increased mortality for only retired men under 64 years. Thus, relative poverty only after retirement may modify this effect, particularly among younger retired men. We also considered that workers who retired younger age may have lost their job after having serious illnesses, because we observed that poor health rate (fair or bad) was higher among younger (47–64 years) retired men (55.1%) than among workers (41.1%). In addition, higher prevalence of cancer and stroke was significantly observed among younger retired men than among workers (data not shown). Although we included perceived health status in the statistical models to eliminate the confounding, residual confounding may still remain, distorting a causal relationship of retirement on mortality. Another possible explanation is that the effect of income on mortality in later life may weaken with aging because additional competitive risks emerge. Collectively, these results indicate that preventing a rapid decline in income only after retirement may prevent the increased mortality risk following retirement. More long-term studies investigating the relationship between income and mortality risk in the elderly, particularly after retirement (e.g., the elderly passed 20 years after retirement), are needed.

In the present study, we found that social support and isolation did not significantly affect the mortality rates of both retired and working men. However, marital status, living alone (for workers), and a lack of participation in social activities (for the retired) were associated with a higher risk of mortality. These findings are supported by a previous study published in 2002 that also analyzed the *Komo-Ise* cohort but did not consider retirement status [15]. A lack of participation in social activities may cause a higher mortality rate after retirement because of decreased social networking in these individuals. A previous study suggested that retirement increased the risk of depressive symptoms in relation to social participation among Japanese older adults [17]. The results of both the present and previous studies demonstrate the importance of maintaining participation in social activities after retirement to prevent increased risk of mortality in the later life. Living alone may also result in a higher mortality risk among male workers. This finding demonstrates the importance of living conditions for workers. Our findings support that retirement is related to the effect of social engagement, the effect of participation in social activities, and the effect of an individual’s living condition on mortality. However, this effect may be heterogeneous. In any case, relationships of family size and health are becoming increasingly important because the number of elderly living alone has been increasing during the past two decades in Japan. In 2015, 11.1% of all households consist of elderly (65 years and older) living alone [12].

Regarding occupational status, self-employed men showed higher crude mortality rates and a significantly higher multiple-adjusted HR than white-collar employees among both working and retired men. Self-employment may be an occupation with high-risk mortality in Japan partly because self-employed individuals get health check-ups less frequently than white-collar employees [18]. Interestingly, the higher hazard of death among self-employed men was not significant among retired men. Our findings suggest that the effect of retirement on mortality may be modified by occupation. Despite this modification, our findings further demonstrate that self-employed workers in later life should be carefully considered for inclusion in epidemiologic studies because of their unique socioeconomic position. Unfortunately, previous studies conducted in Japan using the Japan Standard Occupational Classification have lacked a self-employment job category [9, 21, 22], while a self-employment category was included as an occupational class in studies conducted in European countries [8]. Our findings emphasize that a self-employment category is an important data point and may allow for further elucidation of the unique aspects of this socioeconomic position in future studies.

In addition to the factors mentioned above, white- and blue-collar designations were also used to address socioeconomic position in previous studies conducted in Japan, although it seems that the definition of these categories varies slightly across studies [22–24]. One tool for the standardization of occupation as a socioeconomic position indicator is the Japanese Socioeconomic Classification [25]. Men working in agriculture and forestry had a lower mortality rate than white-collar employees did, although this difference was not significant. We contest that this observation was due to the “healthy worker effect” [26]. However, this finding contradicts a previous report that found a higher mortality rate among Japanese farmers than in other careers [9]. This discrepancy should be addressed in future studies.

This study has several primary limitations. First, we could not collect data on detailed health status of the participants at the time of retirement. For example, some workers included here, particularly men who retired at younger ages (e.g., under 60 years old), might have retired due to serious illness. Conversely, some workers, particularly those who were self-employed, continued working because they were healthier than their retired peers, rendering comparisons more difficult and complex. We recognize the importance of surveying across the lifespan and not only in later life when assessing the reasons for retirement in further social epidemiological studies. Second, timing and age of retirement was unclear in this cohort because we identified retirement status from dual surveys in 1993 and 2000. This resulted in an unclear period after retirement among the deceased. Third, we failed to statistically analyze participant causes of death because the number of deaths by cause was too small to produce reliable estimates. In addition, as income generally decreased with age after retirement, we may have failed to eliminate the confounding effects of income level on age of mortality, even after correcting for age. Fourth, we could not follow the individuals who were working at baseline but retired during the follow-up period. This bias therefore may dismiss the relationship between relative poverty/social relationship and mortality. Finally, our analysis did not have enough statistical power due to the relatively small number of deaths among women. For example, only 3 deaths were noted in 10 years among 52 unmarried women.

## Conclusion

Relative poverty may be related to a higher mortality risk for retired men, but not for working men in middle-aged and elderly community dwelling Japanese. Marital status and living conditions (living alone) were associated with increased risk of mortality among workers. Meanwhile, lack of participation in social activities was associated with increased risk for mortality among retired men. Some employment categories, such as self-employment and working in agriculture and forestry industries, should be considered as risk factors for early death. Our findings highlight that Japan should develop strategies against hyper-aged society with high rates of relative poverty and living alone. Further studies are necessary to better understand the health of middle-aged and elderly population around retirement.

## Abbreviations

HR: Hazard ratio
JPY: Japanese yen
OECD: Organization for Economic Cooperation and Development
SD: Standard deviation
USD: US dollars
